# Differentiating brain metastasis from glioblastoma by time-dependent diffusion MRI

**DOI:** 10.1186/s40644-023-00595-2

**Published:** 2023-08-08

**Authors:** Kiyohisa Kamimura, Yoshiki Kamimura, Tsubasa Nakano, Tomohito Hasegawa, Masanori Nakajo, Chihiro Yamada, Kentaro Akune, Fumitaka Ejima, Takuro Ayukawa, Soichiro Ito, Hiroaki Nagano, Koji Takumi, Masatoyo Nakajo, Hiroyuki Uchida, Kazuhiro Tabata, Takashi Iwanaga, Hiroshi Imai, Thorsten Feiweier, Takashi Yoshiura

**Affiliations:** 1 Department of Advanced Radiological Imaging, Kagoshima University Graduate School of Medical and Dental Sciences, 8-35-1 Sakuragaoka, Kagoshima, 890-8544 Japan; 2 Department of Radiology, Kagoshima University Graduate School of Medical and Dental Sciences, 8-35-1 Sakuragaoka, Kagoshima, 890-8544 Japan; 3 Department of Neurosurgery, Kagoshima University Graduate School of Medical and Dental Sciences, 8-35-1 Sakuragaoka, Kagoshima, 890-8544 Japan; 4 Department of Pathology, Kagoshima University Graduate School of Medical and Dental Sciences, 8-35-1 Sakuragaoka, Kagoshima, 890-8544 Japan; 5 Department of Radiological Technology, Kagoshima University Hospital, 8-35-1 Sakuragaoka, Kagoshima, 890-8544 Japan; 6 Siemens Healthcare K.K., Gate City Osaki West Tower, 1-11-1 Osaki, Shinagawa-Ku, Tokyo, 141-8644 Japan; 7 Siemens Healthcare GmbH, Henkestrasse 127, 91052 Erlangen, Germany

**Keywords:** Diffusion, Glioblastoma, Magnetic resonance imaging, Neoplasm metastasis

## Abstract

**Background:**

This study was designed to investigate the use of time-dependent diffusion magnetic resonance imaging (MRI) parameters in distinguishing between glioblastomas and brain metastases.

**Methods:**

A retrospective study was conducted involving 65 patients with glioblastomas and 27 patients with metastases using a diffusion-weighted imaging sequence with oscillating gradient spin-echo (OGSE, 50 Hz) and a conventional pulsed gradient spin-echo (PGSE, 0 Hz) sequence. In addition to apparent diffusion coefficient (ADC) maps from two sequences (ADC_50Hz_ and ADC_0Hz_), we generated maps of the ADC change (cADC): ADC_50Hz_ − ADC_0Hz_ and the relative ADC change (rcADC): (ADC_50Hz_ − ADC_0Hz_)/ ADC_0Hz_ × 100 (%).

**Results:**

The mean and the fifth and 95th percentile values of each parameter in enhancing and peritumoral regions were compared between glioblastomas and metastases. The area under the receiver operating characteristic curve (AUC) values of the best discriminating indices were compared. In enhancing regions, none of the indices of ADC_0Hz_ and ADC_50Hz_ showed significant differences between metastases and glioblastomas. The mean cADC and rcADC values of metastases were significantly higher than those of glioblastomas (0.24 ± 0.12 × 10^−3^mm^2^/s vs. 0.14 ± 0.03 × 10^−3^mm^2^/s and 23.3 ± 9.4% vs. 14.0 ± 4.7%; all *p* < 0.01). In peritumoral regions, no significant difference in all ADC indices was observed between metastases and glioblastomas. The AUC values for the mean cADC (0.877) and rcADC (0.819) values in enhancing regions were significantly higher than those for ADC_0Hz_^5th^ (0.595; all *p* < 0.001).

**Conclusions:**

The time-dependent diffusion MRI parameters may be useful for differentiating brain metastases from glioblastomas.

## Background

Glioblastomas and brain metastases are the most common intra-axial brain tumors in adults. Since these two tumor types are substantially different with respect to clinical workup and therapeutic strategies [1], their pretreatment differentiation is essential. Magnetic resonance imaging (MRI) is the modality of choice for the preoperative imaging assessment of brain tumors. However, differentiating brain metastases from glioblastomas based on conventional MRI may be difficult, as their findings are sometimes similar [2]. Diffusion-weighted imaging (DWI) reflects the Brownian motion of water molecules. DWI and quantitative measurement of the apparent diffusion coefficient (ADC) add valuable information regarding microstructures of tumor tissues to the conventional MRI findings. A weak to moderate inverse relationship was observed between ADC values and tumor cellularity [3–5]. Studies have shown that ADC values can help differentiate between certain types of brain tumors: malignant and benign meningiomas; high- and low-grade gliomas; and glioblastomas and primary central nervous system lymphomas [6–8]. However, the usefulness of the ADC values in differentiating brain metastases from glioblastomas remains controversial [9–12].

Diffusion time is an essential parameter of a DWI sequence that determines the duration over which water diffusion is assessed [13]. The ADC value matches the true diffusion coefficient only when diffusion is free (Gaussian), and this diffusion coefficient does not depend on the b-values or the diffusion time. However, when diffusion in tissues is not Gaussian, it depends on the interactions of molecules with spatial barriers, such as fibers and cell membranes (restricted diffusion). In the presence of restricted diffusion, the ADC values increase with decreasing diffusion time [14–17]. In conventional DWI based on pulsed gradient spin-echo (PGSE), high b-value is used to probe restricted diffusion, leading to a long diffusion time due to the limited maximum achievable gradient strength in clinical MRI systems [18, 19]. Recently, the oscillating gradient spin-echo (OGSE) method has become available on clinical MRI scanner [14]. This method can shorten the diffusion time by substituting the long diffusion sensitizing gradients used in PGSE methods with rapid oscillation gradients. DWI with the OGSE method enables to shorten the diffusion times and thus allows the exploration of the ADC diffusion time dependencies at the short diffusion time regime, which are inaccessible using the PGSE method alone. Time-dependent diffusion MRI is considered to provide more detailed information regarding tissue microstructure and has currently been tested in clinical settings to evaluate normal brains [14, 20], intracranial epidermoid cysts [21], head and neck tumors [22], and brain tumors [23]. It is conceivable that brain metastases originating from outside the central nervous system substantially differ from glioblastomas in terms of microstructure. In particular, epithelial tumors in the body are characterized by cell–cell adhesion that can narrow the extracellular space. This microstructural feature may be associated with a higher volume fraction of the intracellular space, where water molecular diffusion is restricted by the cell membrane and a clear diffusion time dependence of water diffusion is present [24]. We hypothesized that time-dependent diffusion MRI can detect such microstructural feature of brain metastasis, providing a valuable clue to the differentiation from glioblastoma. Thus, our purpose was to investigate the usefulness of the time-dependent diffusion MRI parameters obtained using the OGSE and PGSE methods in differentiating brain metastases from glioblastomas.

## Materials and methods

### Patients

Our Institutional Review Board approved this retrospective study (approval no. 220126) and waived the need for written informed consent. The inclusion criteria were consecutive patients with pathologically proven glioblastoma or brain metastasis who underwent MRI including OGSE and PGSE sequences as a part of routine pretreatment assessments between January 2019 and September 2022 at our institution. All glioblastomas were diagnosed based on an integrated diagnosis combining histology and a glioma-tailored next-generation sequencing panel developed in our institution [25], and fulfilled the World Health Organization classification of 2021 [26]. The exclusion criteria were (a) lack of preoperative MRI, including DWI with both OGSE and PGSE sequences; (b) poor image quality; (c) masses smaller than 1 cm; (d) previous surgical resection or irradiation; or (e) lack of contrast-enhancing lesions.

In patients with multiple lesions, the largest mass was examined by MRI.

**Fig. 1:**
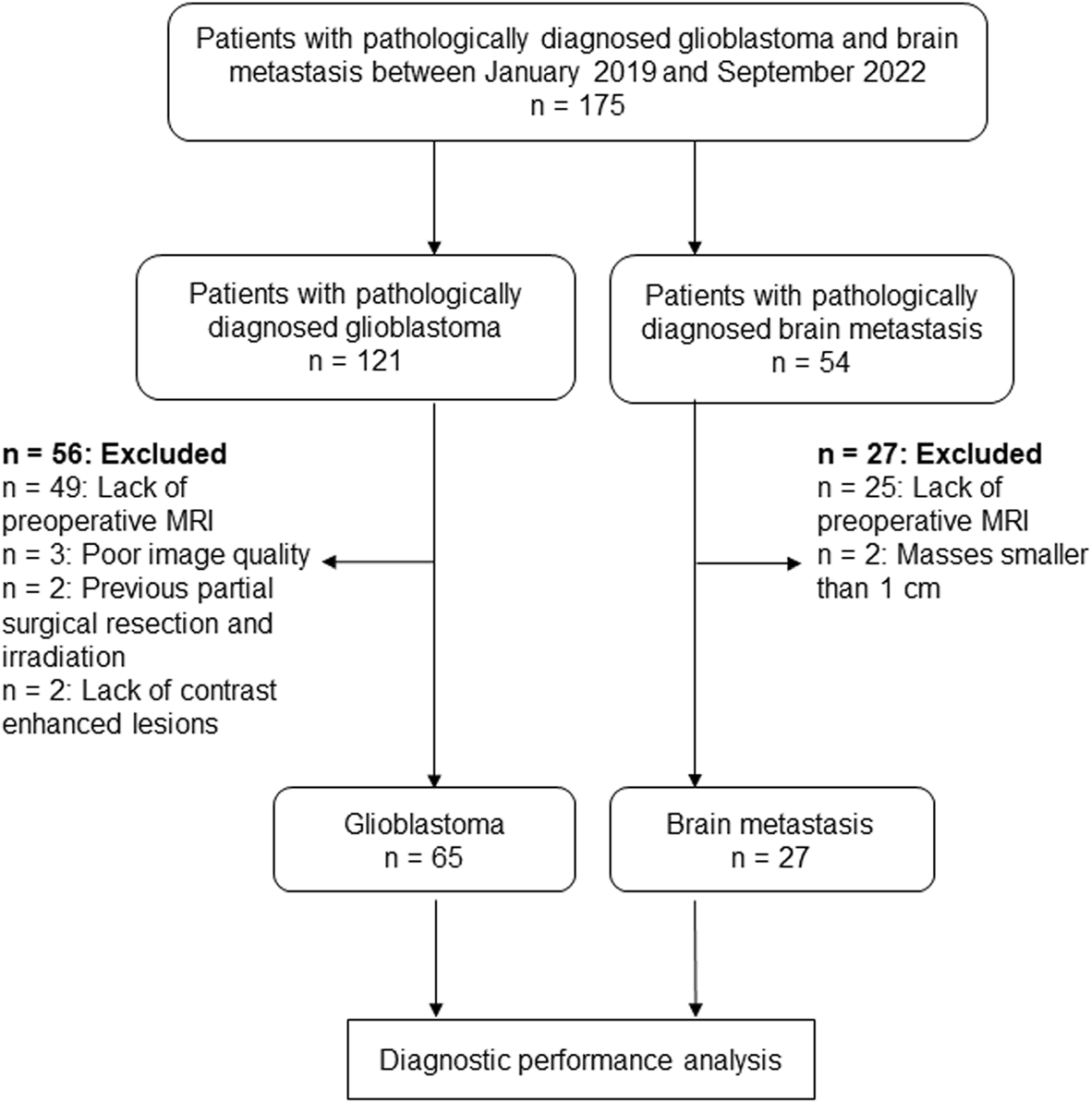
The study chart shows the inclusion and exclusion criteria and pathways for eligible patients in this study

In this study, 175 consecutive patients (121 with glioblastomas and 54 with brain metastases) were considered. Among them, 83 were excluded owing to the absence of preoperative MRI including both OGSE and PGSE DWI scans (49 with glioblastomas and 25 with brain metastases), masses smaller than 1 cm (two with brain metastases), poor image quality caused by artifacts in the DWIs (three with glioblastomas), previous surgical resection or irradiation (two with glioblastomas), or lack of contrast-enhancing lesions (two with glioblastomas). Thus, 92 patients (56 men and 36 women; age range, 15–91 years; mean age, 69 ± 12 years) met the inclusion criteria. Sixty-five patients with isocitrate dehydrogenase-wildtype glioblastomas (37 men and 28 women; age range 15–91 years; mean age, 69 ± 13 years) and 27 with brain metastases (19 men and eight women; age range 47–80 years; mean age, 68 ± 10 years; 15 from lung cancer, four from breast cancer, three from colon cancer, two from gastric cancer, one from bladder cancer, one from submandibular cancer, and one from spindle cell sarcoma) were finally analyzed (Fig. 1). Eight patients had multiple brain metastases. No patient was treated for brain tumor before MRI. Histopathological confirmation was obtained based on total or partial surgical resection in all patients. Table 1 shows the characteristics of the patients. Sixty-five patients were diagnosed with glioblastomas, and 27 were diagnosed with brain metastases. No significant differences in age and sex were observed between patients with glioblastomas and those with brain metastases.

**Table 1:** Characteristics of the patients

Patients’ characteristics	Total (*n* = 92)	Glioblastoma (*n* = 65)	Brain metastasis (*n* = 27)	*p* Value
Age (y)	69 ± 12	69 ± 13	68 ± 10	0.49^a^
No. of men	56 (60.9%)	37 (56.9%)	19 (70.4%)	0.23^b^

Statistical tests used: ^a^Mann–Whitney *U* test, ^b^chi-square test

### MRI acquisition

**Fig. 2:**
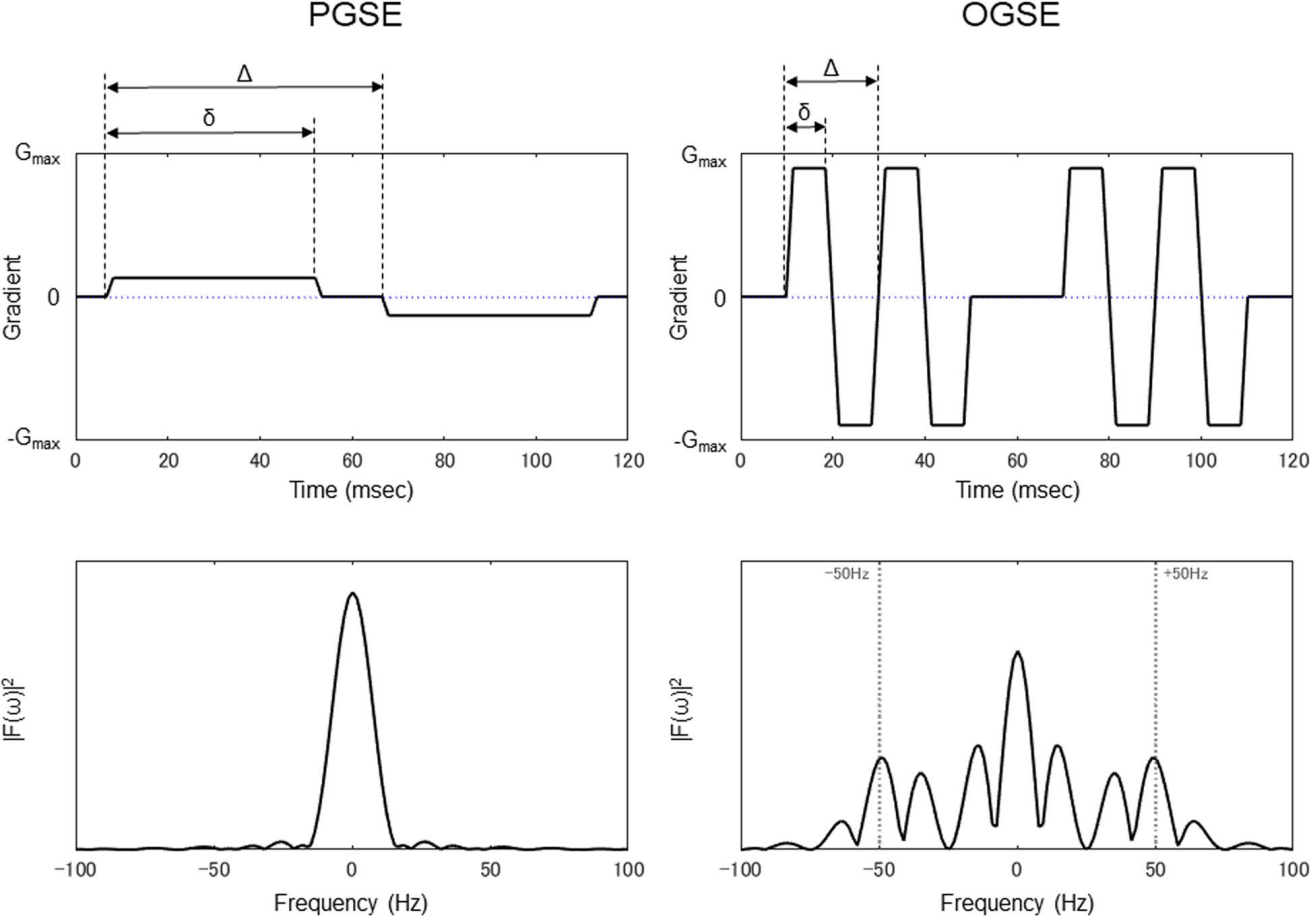
Schematic representation of the diffusion gradient waveforms, (top line) and their corresponding diffusion encoding spectrums, |F(ω)|^2^, (bottom line) for pulsed gradient spin-echo (PGSE) (left) and oscillating gradient spin-echo (OGSE) (right). A 180° RF pulse is applied to the center of the gradient pair; therefore, the second gradient waveform acts as the opposite polarity. Δ_eff_ = Δ − δ/3. Δ_eff_, effective diffusion time; *Δ*, diffusion gradient separation; *δ*, diffusion gradient pulse duration

All patients were scanned on a 3 T MR scanner (MAGNETOM Prisma; Siemens Healthcare; maximum gradient amplitude = 80 mT/m, maximum slew rate = 200 T/m/s for each gradient axis with a 20-channel head radiofrequency receive coil. DWI was performed with research sequences for the OGSE DWI using b-values of 0 s/mm^2^ (number of excitation: 1) and 1,500 s/mm^2^ (number of excitations: 4) and three diffusion encoding directions. OGSE diffusion encoding used trapezoid-sine waveforms [27] with an effective diffusion time (Δ_eff_) of 7.1 ms (frequency = 50 Hz; diffusion gradient pulse duration [δ] = 8.5 ms). The Δ_eff_ for the PGSE encoding was 44.5 ms (frequency = 0 Hz; diffusion gradient separation [Δ] = 59.8 ms; δ = 46.1 ms). The two sequences shared the following parameters: repetition time (TR), 4,600 ms; echo time (TE), 120 ms; field of view (FOV), 230 × 230 mm^2^; matrix size, 72 × 72; number of slices, 24; and slice thickness, 5 mm. The acquisition times for PGSE DWI and OGSE DWI were 1 min and 13 s, and 1 min and 19 s, respectively. The pulse sequence diagrams for OGSE and PGSE are shown in Fig. 2.

Precontrast 2D fluid-attenuated inversion recovery (FLAIR) images were acquired using the following parameters: TR, 9,000 ms; TE, 121 ms; TI, 2,530 ms; number of excitations, 1; matrix, 307 × 384 (reconstructed to 768 × 768); number of slices, 24; slice thickness, 5 mm; interslice gap, 1 mm; FOV, 230 × 230 mm^2^; and scan time, 2 min and 6 s, and postcontrast 2D T1-weighted spin-echo images were also acquired using the following parameters: TR, 520 ms; TE, 12 ms; number of excitations, 1; matrix, 269 × 384 (reconstructed to 768 × 768); number of slices, 24; slice thickness, 5 mm; interslice gap, 1 mm; FOV, 230 × 230 mm^2^; and scan time, 2 min and 26 s. Then, these images were used for anatomical reference during the delineation of the region of interests (ROIs). In addition, our routine imaging for the central nervous system region included the following precontrast sequences (Table 2): 2D T1-weighted spin-echo imaging, 2D T2-weighted turbo spin-echo imaging, and 3D susceptibility-weighted imaging. These sequences were not actively used in this study, although precontrast T1-weighted images were used to confirm contrast enhancement.

**Table 2:** Imaging parameters of pre and postcontrast conventional MRI sequences

	**Precontrast 2D T1-weighted imaging**	**2D T2-weighted imaging**	**2D fluid-attenuated inversion recovery imaging**	**3D susceptibility-weighted imaging**	**Postcontrast** **2D T1-weighted imaging**
Sequence	2D SE	2D TSE	2D IR-TSE	3D FLASH	2D SE
TR (ms)	520	4000	9000	28	520
TE (ms)	12	91	121	20	12
TI (ms)	N/A	N/A	2530	N/A	N/A
FA (degree)	70/180	150	120	15	70/180
Bandwidth (Hz/pixel)	181	199	130	120	181
Number of excitations	1	1	1	1	1
Turbo factor	N/A	9	25	N/A	N/A
Acceleration factor	N/A	2	2	2	N/A
FOV (mm)	230	230	230	230	230
Matrix	269 × 384	380 × 448	307 × 384	240 × 320	269 × 384
Thickness (mm)	5	5	5	2.5	5
Intersection gap (mm)	1	1	1	N/A	1
Acquisition time (s)	148	80	126	174	148

### Delineation of the ROI

Two independent radiologists (T.H. and Y.K., with 7 and 3 years of radiological experience, respectively), who were blinded to the patients’ clinical and pathological information, performed the ROI analysis using a commercially available software (Vitrea; Canon Medical Systems Corporation). ROIs were placed manually on a postcontrast T1-weighted image with the largest tumor diameter, including enhancing region and avoiding necrosis and fluid, such as nonenhancing regions in the tumor, and on the corresponding FLAIR image, including nonenhancing peritumoral regions with a FLAIR high signal intensity. The ROI size of the enhancing and the nonenhancing peritumoral regions was 653 ± 488 mm^2^ (range, 113–2,383 mm^2^) and 622 ± 607 mm^2^ (28–2,817 mm^2^), respectively, for glioblastomas, and 437 ± 290 mm^2^ (252–894 mm^2^) and 925 ± 773 mm^2^ (59–3,495 mm^2^), respectively, for brain metastases.

### Processing

ADC values were calculated, as follows:

1 $$\mathrm{ADC }=\mathrm{ ln}({\mathrm{S}}_{0}/{\mathrm{S}}_{1})/(\mathrm{b}1 -\mathrm{ b}0),$$

where S_0_ and S_1_ are the signal intensities measured from DWI obtained using lower (b0) and higher (b1) b-values.

Researchers have evaluated the diffusion time dependence of ADC by calculating the change in the ADC between the OGSE and PGSE sequences and its ratio to the ADC derived from PGSE [22, 23]. We used both the ADC change (cADC) and the relative ADC change (rcADC) between OGSE and PGSE. cADC and rcADC maps were generated using the pixel-by-pixel calculation method, using the following formulas:

2 $$\mathrm{cADC }= {\mathrm{ADC}}_{50\mathrm{Hz}} - {\mathrm{ADC}}_{0\mathrm{Hz}},$$

3 $$\mathrm{rcADC }= ({\mathrm{ADC}}_{50\mathrm{Hz}} - {\mathrm{ADC}}_{0\mathrm{Hz}})/{\mathrm{ADC}}_{0\mathrm{Hz}} \times 100 (\mathrm{\%}),$$

where ADC_50Hz_ and ADC_0Hz_ are the ADC values obtained using a DWI sequence with OGSE (50 Hz) and a conventional PGSE (0 Hz) sequence, respectively.

### ROI-based measurement

The ADC maps were coregistered with the postcontrast T1-weighted images using the rigid body registration on Vitrea. The ROIs of the enhancing region drawn on the postcontrast T1-weighted images and the ROIs of the peritumoral region drawn on the FLAIR images were duplicated on each ADC map and cADC and rcADC maps. Using the ROIs, the mean ADC_0Hz_ (ADC_0Hz_^mean^), ADC_50Hz_ (ADC_50Hz_^mean^), cADC (cADC^mean^), and rcADC (rcADC^mean^) were calculated for the entire ROI. In addition, the fifth and 95th percentiles of the ADC_0Hz_ (ADC_0Hz_^5th^ and ADC_0Hz_^95th^), ADC_50Hz_ (ADC_50Hz_^5th^ and ADC_50Hz_^95th^), cADC (cADC^5th^ and cADC^95th^), and rcADC (rcADC^5th^ and rcADC^95th^) were calculated, with these being considered to be representative of the lowest and highest robust values, respectively [28].

### Statistical analysis

The D’Agostino–Pearson normality test was used to verify the normality of the data obtained. The mean age was compared between those who had brain metastases and those who had glioblastoma using the Mann–Whitney *U* test, and the gender distribution was compared using the chi-square test. The interobserver agreement on parametric measures between the two observers was analyzed by computing the intraclass correlation coefficient (ICC). ICCs over 0.74 indicate excellent agreement [29]. Measurements taken by both observers for each patient were averaged for more in-depth analysis. The paired-*t* test was used for comparison of the ADC values with different diffusion times. The unpaired *t* test or Mann–Whitney *U* test was used for the comparison of the mean and the fifth and 95th percentiles of ADC_0Hz_, ADC_50Hz_, cADC, and rcADC values. The area under the receiver operating characteristic curve (AUC) of each parameter was calculated. Sensitivity and specificity were obtained using a threshold criterion to maximize the Youden index. Differences in diagnostic performance were investigated with AUCs. In the enhancing regions, the most effective indices were determined for each ADC_0Hz_, ADC_50Hz_, cADC, and rcADC. The AUCs of the most effective indices were compared using DeLong’s test. Bonferroni correction was done to accommodate multiple comparisons. Statistical analyses were carried out using commercially available software packages (MedCalc, version 15.10.0; MedCalc statistical software). *P* values smaller than 0.05 were used to indicate statistical significance.

## Results

**Fig. 3:**
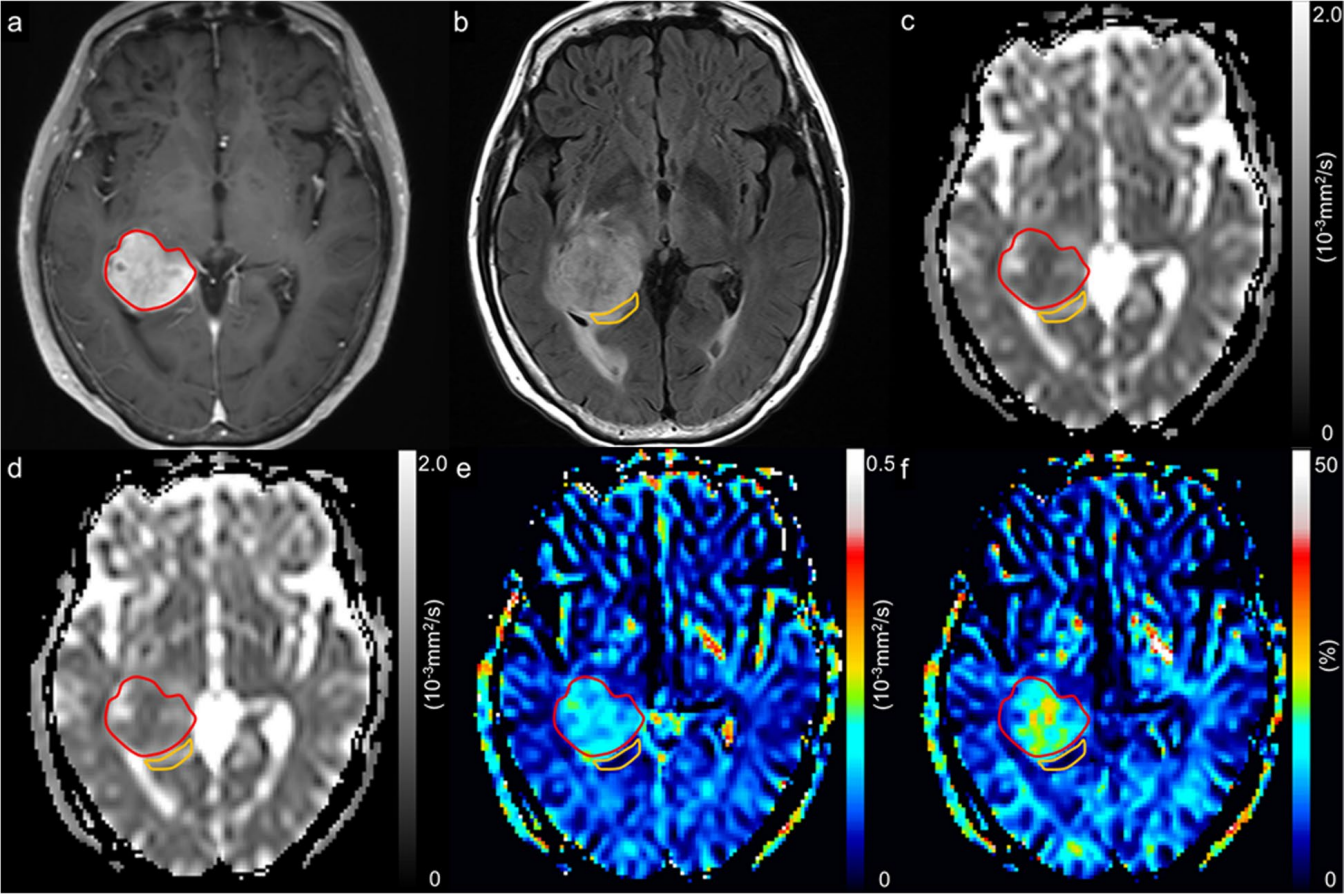
A 74-year-old woman with glioblastoma, isocitrate dehydrogenase-wildtype, grade 4. A contrast-enhanced T1-weighted image with a region of interest of the enhancing region (red line) (**a**), a FLAIR image with a region of interest of peritumoral region (orange line) (**b**), an apparent diffusion coefficient (ADC) map derived from pulsed gradient spin-echo (PGSE) DWI at an effective diffusion time (Δ_eff_) of 44.5 ms (**c**), an ADC map derived from oscillating gradient spin-echo (OGSE) DWI at an Δ_eff_ of 7.1 ms (**d**), and maps of ADC change between PGSE DWI and OGSE DWI (cADC) (**e**) and relative ADC change between PGSE DWI and OGSE DWI (rcADC) (**f**). The ADC values in the tumor appear higher at short Δ_eff_ values than at long Δ_eff_ setting. Small changes in cADC and rcADC are noted between the OGSE and PGSE sequences in the tumor

**Fig. 4:**
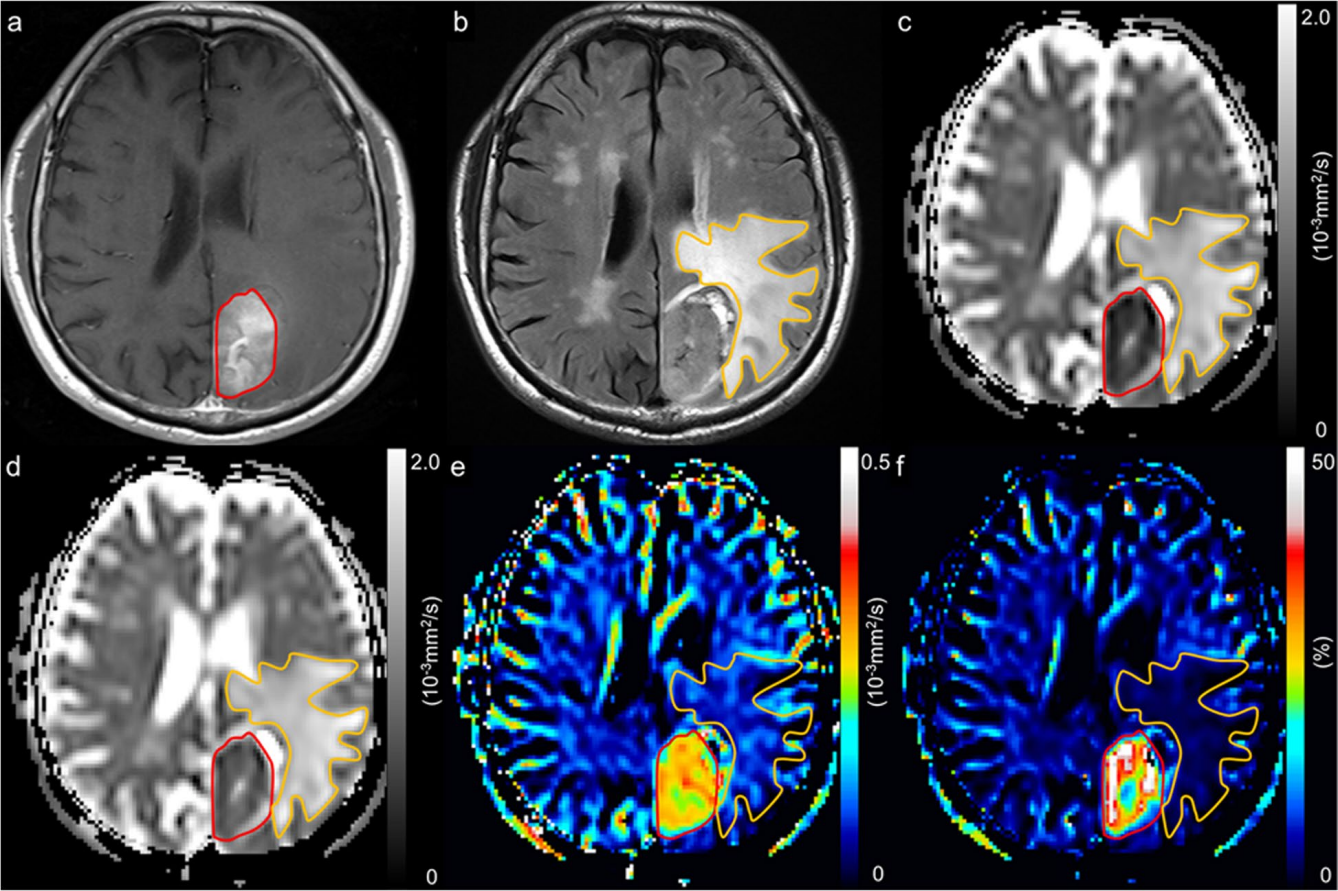
A 69-year-old man with a brain metastasis from colon cancer. A contrast-enhanced T1-weighted image with a region of interest of the enhancing region (red line) (**a**), a FLAIR image with a region of interest of peritumoral region (orange line) (**b**), an apparent diffusion coefficient (ADC) map derived from pulsed gradient spin-echo (PGSE) DWI at an effective diffusion time (Δ_eff_) of 44.5 ms (**c**), an ADC map derived from oscillating gradient spin-echo (OGSE) DWI at an Δ_eff_ of 7.1 ms (**d**), and maps of ADC change between PGSE DWI and OGSE DWI (cADC) (**e**) and relative ADC change between PGSE DWI and OGSE DWI (rcADC) (**f**). The ADC values in the tumor appear higher at short Δ_eff_ values than at long Δ_eff_ setting. Large changes in cADC and rcADC are noted between the OGSE and PGSE sequences in the tumor

The representative diffusion parametric maps of glioblastoma and brain metastasis are shown in Figs. 3 and 4.

### Interobserver agreement

The ICCs and 95% confidence intervals for each parameter are shown in Table 3. All parameters showed an excellent agreement.

**Table 3:** The intraclass correlation coefficients and 95% confidence intervals for ADC_0Hz_^mean^, ADC_0Hz_^5th^, ADC_0Hz_^95th^, ADC_50Hz_^mean^, ADC_50Hz_^5th^, ADC_50Hz_^95th^, cADC^mean^, cADC^5th^, cADC^95th^, rcADC^mean^, rcADC^5th^, and rcADC^95th^ of the enhancing and peritumoral regions

Parameters	Intraclass correlation coefficients (95% confidence intervals)	
	**Enhancing region**	**Peritumoral region**
ADC_0Hz_^mean^	0.961 (0.943–0.974)	0.993 (0.989–0.995)
ADC_0Hz_^5th^	0.905 (0.860–0.936)	0.969 (0.953–0.979)
ADC_0Hz_^95th^	0.862 (0.799–0.906)	0.987 (0.980–0.991)
ADC_50Hz_^mean^	0.967 (0.950–0.978)	0.992 (0.988–0.995)
ADC_50Hz_^5th^	0.908 (0.865–0.938)	0.962 (0.943–0.974)
ADC_50Hz_^95th^	0.889 (0.838–0.925)	0.990 (0.985–0.993)
cADC^mean^	0.974 (0.963–0.982)	0.995 (0.993–0.997)
cADC^5th^	0.825 (0.748–0.880)	0.982 (0.973–0.988)
cADC^95th^	0.981 (0.971–0.987)	0.992 (0.988–0.995)
rcADC^mean^	0.968 (0.953–0.979)	0.998 (0.997–0.998)
rcADC^5th^	0.868 (0.808–0.910)	0.983 (0.975–0.989)
rcADC^95th^	0.952 (0.929–0.968)	0.995 (0.992–0.997)

### Diffusion indices of brain metastases and glioblastomas

**Fig. 5:**
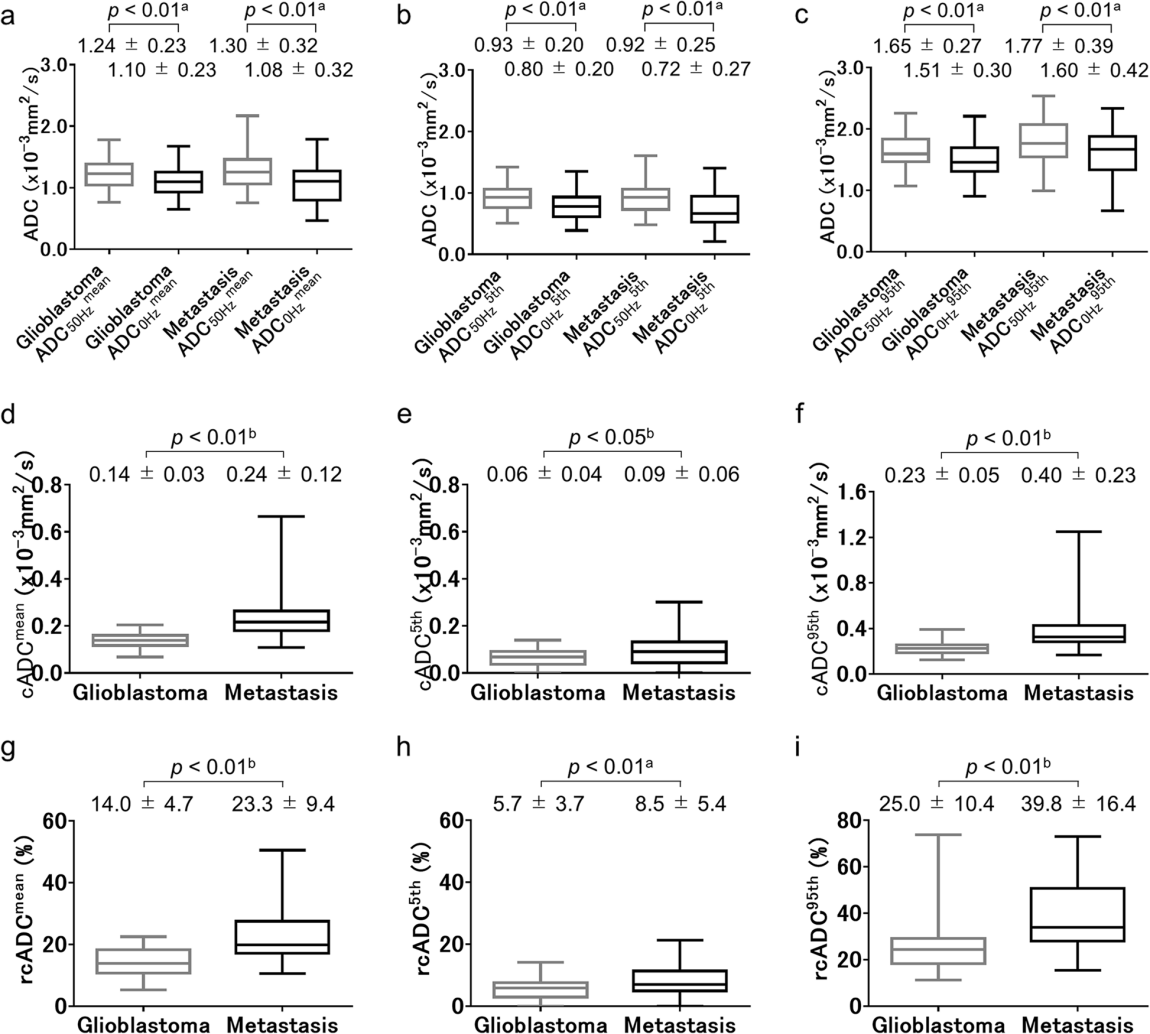
Box-whisker plots of ADC_0Hz_^mean^ and ADC_50Hz_^mean^ (**a**), ADC_0Hz_^5th^ and ADC_50Hz_^5th^ (**b**), and ADC_0Hz_^95th^ and ADC_50Hz_^95th^ (**c**) of enhancing regions for glioblastomas and brain metastases. For each tumor, each index for ADC_50Hz_ was significantly higher than the corresponding index for ADC_0Hz_ (each *p* < 0.01, respectively) (**a**–**c**). Box-whisker plots of cADC^mean^ (**d**), cADC^5th^ (**e**), and cADC^95th^ (**f**) of enhancing regions for glioblastomas and brain metastases. Each index for cADC was significantly higher in brain metastases than in glioblastomas (each *p* < 0.01, respectively). Box-whisker plots of rcADC^mean^ (**g**), rcADC^5th^ (**h**), and rcADC.^95th^ (**i**) of enhancing regions for glioblastomas and brain metastases. Each index for rcADC was significantly higher in brain metastases than in glioblastomas (each *p* < 0.01, respectively). Statistical tests used: _a_paired-*t* test, _b_Mann–Whitney *U* test

The ADC_0Hz_^mean^, ADC_0Hz_^5th^, ADC_0Hz_^95th^, ADC_50Hz_^mean^, ADC_50Hz_^5th^, and ADC_50Hz_^95th^ values of the enhancing regions for glioblastomas and brain metastases are tabulated in Fig. 5a–c. For both tumors, all three indices for ADC_50Hz_ were significantly higher than those of ADC_0Hz_ (all *p* < 0.01, respectively) (Fig. 5a–c). No significant difference was observed between brain metastases and glioblastomas in any of the three indices of ADC_0Hz_ and ADC_50Hz_ (Fig. 5a–c). The cADC^mean^ (*p* < 0.01), cADC^5th^ (*p* < 0.05), cADC^95th^ (*p* < 0.01), rcADC^mean^ (*p* < 0.01), rcADC^5th^ (*p* < 0.01), and rcADC^95th^ (*p* < 0.01) values were significantly higher for brain metastases than for glioblastomas (Fig. 5d–i).

**Fig. 6:**
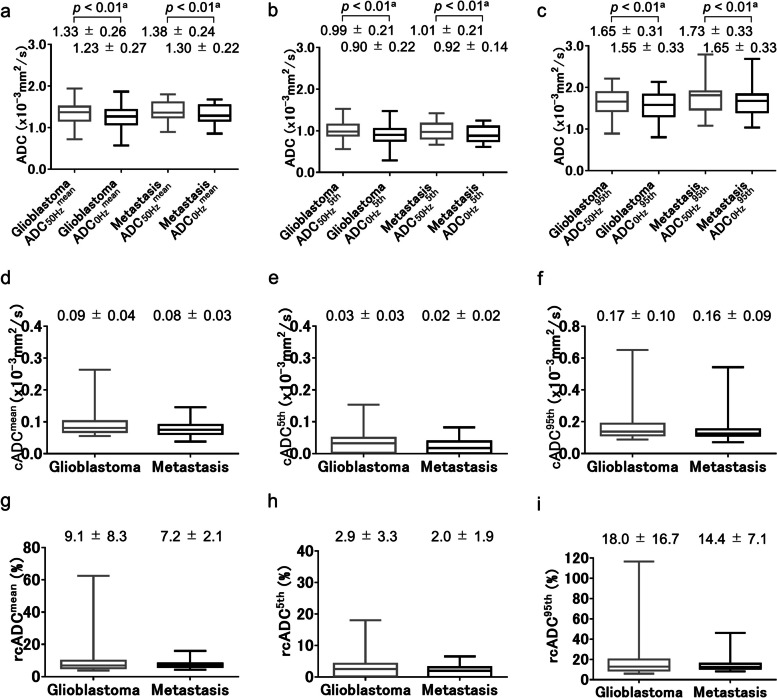
Box-whisker plots of ADC_0Hz_^mean^ and ADC_50Hz_^mean^ (**a**), ADC_0Hz_^5th^ and ADC_50Hz_^5th^ (**b**), and ADC_0Hz_^95th^ and ADC_50Hz_^95th^ (**c**) of peritumoral regions for glioblastomas and brain metastases. For each tumor, each index for ADC_50Hz_ was significantly higher than the corresponding index for ADC_0Hz_ (each *p* < 0.01, respectively) (**a**–**c**). Box-whisker plots of cADC^mean^ (**d**), cADC^5th^ (**e**), and cADC^95th^ (**f**) of peritumoral regions for glioblastomas and brain metastases. Box-whisker plots of rcADC^mean^ (**g**), rcADC^5th^ (**h**), and rcADC.^95th^ (**i**) of peritumoral regions for glioblastomas and brain metastases. No significant difference in any of the three indices of ADC_0Hz_, ADC_50Hz_, cADC, and rcADC was observed between brain metastases and glioblastomas (Fig. 6a–i). Statistical tests used: _a_paired-*t* test, _b_Mann–Whitney *U* test

The ADC_0Hz_^mean^, ADC_0Hz_^5th^, ADC_0Hz_^95th^, ADC_50Hz_^mean^, ADC_50Hz_^5th^, and ADC_50Hz_^95th^ values of the peritumoral regions for glioblastomas and brain metastases are shown in Fig. 6a–c. For both tumors, all three indices for ADC_50Hz_ were significantly higher than those for ADC_0Hz_ (all *p* < 0.01, respectively) (Fig. 6a–c). No significant difference in any of the three indices of ADC_0Hz_, ADC_50Hz_, cADC, and rcADC was observed between brain metastases and glioblastomas (Fig. 6a–i).

### Diagnostic performance in differentiating brain metastases from glioblastoma

**Fig. 7:**
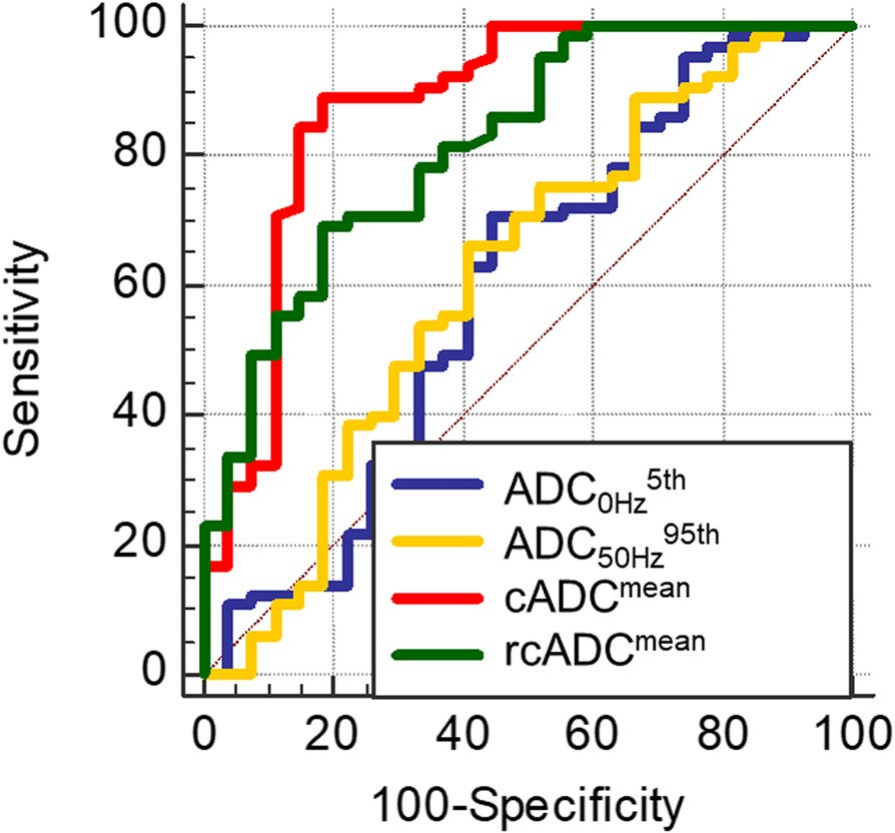
Receiver operating characteristic curves of the most effective indices for ADC_0Hz_^5th^, ADC_50Hz_^95th^, cADC^mean^, and rcADC^mean^

The results of receiver operating characteristic (ROC) curve analyses are shown in Table 4. The ROC curve analysis showed significance for cADC^mean^, cADC^95th^, rcADC^mean^, rcADC^5th^, and rcADC^95th^ (AUC = 0.877, 0.865, 0.819, 0.652, and 0.796; respectively; *p* < 0.01, *p* < 0.01, *p* < 0.01, *p* = 0.02, and *p* < 0.01; respectively), and their accuracy in diagnosing glioblastoma was 87.0%, 85.9%, 72.8%, 64.1%, and 79.3%; respectively. The most effective indices for the ADC_0Hz_, ADC_50Hz_, cADC, and rcADC were ADC_0Hz_^5th^, ADC_50Hz_^95th^, cADC^mean^, and rcADC^mean^, respectively. As shown in Table 5, pairwise comparisons of the AUC of ROC curves among those most effective indices revealed that the AUC of the cADC^mean^ was significantly greater than those of ADC_0Hz_^5th^ (*p* < 0.001) and ADC_50Hz_^95th^ (*p* = 0.004) and that the AUC of the rcADC^mean^ was significantly greater than that of ADC_0Hz_^5th^ (*p* < 0.001). No other comparisons of the AUCs revealed significant differences. The ROC curves for the ADC_0Hz_^5th^, ADC_50Hz_^95th^, cADC^mean^, and rcADC^mean^ are shown in Fig. 7.

**Table 4:** The AUC, optimal threshold, sensitivity, specificity, and accuracy for ADC_0Hz_^mean^, ADC_0Hz_^5th^, ADC_0Hz_^95th^, ADC_50Hz_^mean^, ADC_50Hz_^5th^, ADC_50Hz_^95th^, cADC^mean^, cADC^5th^, cADC^95th^, rcADC^mean^, rcADC^5th^, and rcADC^95th^ of the enhancing and peritumoral regions to differentiate brain metastases from glioblastomas

Parameter	AUC (95% CI)	*p* Value	Threshold value	Sensitivity (%)	Specificity (%)	Accuracy (%)
Enhancing region						
ADC_0Hz_^mean^	0.527 (0.420–0.632)	0.71	0.800 (× 10^–3^ mm^2^/s)	80.8	25.9	71.7
ADC_0Hz_^5th^	0.595 (0.487–0.696)	0.19	0.683 (× 10^–3^ mm^2^/s)	70.8	55.6	66.3
ADC_0Hz_^95th^	0.592 (0.485–0.694)	0.20	1.659 (× 10^–3^ mm^2^/s)	73.8	51.9	67.4
ADC_50Hz_^mean^	0.544 (0.437–0.649)	0.53	1.350 (× 10^–3^ mm^2^/s)	72.3	44.4	64.1
ADC_50Hz_^5th^	0.526 (0.419–0.631)	0.71	0.664 (× 10^–3^ mm^2^/s)	96.9	18.5	73.9
ADC_50Hz_^95th^	0.615 (0.508–0.714)	0.10	1.704 (× 10^–3^ mm^2^/s)	66.2	59.3	64.1
cADC^mean^	0.877 (0.793–0.937)	< 0.01	0.174 (× 10^–3^ mm^2^/s)	89.2	81.5	87.0
cADC^5th^	0.630 (0.523–0.729)	0.06	0.081 (× 10^–3^ mm^2^/s)	67.7	59.3	65.2
cADC^95th^	0.865 (0.778–0.927)	< 0.01	0.278 (× 10^–3^ mm^2^/s)	87.7	81.5	85.9
rcADC^mean^	0.819 (0.724–0.891)	< 0.01	16.8 (%)	69.2	81.5	72.8
rcADC^5th^	0.652 (0.546–0.749)	0.02	6.65 (%)	63.1	66.7	64.1
rcADC^95th^	0.796 (0.700–0.873)	< 0.01	30.6 (%)	84.6	66.7	79.3
Peritumoral region						
ADC_0Hz_^mean^	0.566 (0.459–0.669)	0.30	1.156 (× 10^–3^ mm^2^/s)	40.0	81.5	52.2
ADC_0Hz_^5th^	0.509 (0.402–0.615)	0.89	0.669 (× 10^–3^ mm^2^/s)	18.5	96.3	41.3
ADC_0Hz_^95th^	0.559 (0.452–0.662)	0.37	1.615 (× 10^–3^ mm^2^/s)	56.9	59.3	57.6
ADC_50Hz_^mean^	0.558 (0.450–0.661)	0.38	1.242 (× 10^–3^ mm^2^/s)	38.5	77.8	50.0
ADC_50Hz_^5th^	0.509 (0.403–0.615)	0.89	1.267 (× 10^–3^ mm^2^/s)	95.4	14.8	71.7
ADC_50Hz_^95th^	0.559 (0.452–0.662)	0.37	1.691 (× 10^–3^ mm^2^/s)	55.4	63.0	57.6
cADC^mean^	0.598 (0.491–0.699)	0.14	0.072 (× 10^–3^ mm^2^/s)	69.2	48.1	63.0
cADC^5th^	0.580 (0.473–0.682)	0.21	0.038 (× 10^–3^ mm^2^/s)	46.2	77.8	55.4
cADC^95th^	0.569 (0.462–0.672)	0.30	0.140 (× 10^–3^ mm^2^/s)	49.2	70.4	55.4
rcADC^mean^	0.507 (0.401–0.613)	0.91	9.0 (%)	70.8	3.7	51.1
rcADC^5th^	0.568 (0.460–0.671)	0.29	2.6 (%)	47.7	70.4	54.3
rcADC^95th^	0.506 (0.399–0.612)	0.92	8.7 (%)	24.6	96.3	45.7

**Table 5:** Pairwise comparison of the AUCs among the ADC_0Hz_^5th^, ADC_50Hz_^95th^, cADC^mean^, and rcADC^mean^ of the enhancing regions

Parameter	rcADC^mean^	cADC^mean^	ADC_50Hz_^95th^
ADC_0Hz_^5th^			
DBE	0.224	0.283	0.020
*p*	< 0.001	< 0.001	0.877
ADC_50Hz_^95th^			
DBE	0.204	0.263	
*p*	0.042	0.004	
cADC^mean^			
DBE	0.059		
*p*	0.179		

*DBE* Difference between areas

## Discussion

This study revealed no significant difference between brain metastases and glioblastomas in ADCs measured using short (7.1 ms) and long (44.5 ms) effective diffusion times, whereas both the change and relative change of ADC (cADC and rcADC, respectively) were significantly higher in brain metastases than in glioblastomas. Moreover, the ADC change, particularly the cADC^mean^, showed a good performance in differentiating brain metastases from glioblastomas, suggesting the clinical usefulness of time-dependent diffusion MRI for this purpose.

Time-dependent diffusion MRI using OGSE and PGSE DWI sequences has drawn increasing attention among the medical community of oncological imaging. Previously, Iima et al. investigated the use of time-dependent diffusion MRI in distinguishing malignant head and neck tumors from benign ones, involving 56 patients [22]. They found that the relative change in ADC between short (4.3 ms) and long (82.6 ms) effective diffusion times was significantly higher in malignant head and neck tumors than benign ones. Maekawa et al. used two effective diffusion times (6.5 ms and 32.5 ms) and examined 21 brain tumors using time-dependent diffusion MRI and showed that both the ADC change and the relative ADC change were significantly higher for high-grade tumors than for low-grade tumors [23]. Wu et al. used time-dependent MRI to examine the microstructures of 48 prostate cancers [30]. They reported that a higher histopathologic grade was significantly correlated with higher intracellular volume fraction and cellularity derived using a two-compartment diffusion model [31], and that cellularity achieved high performance in discriminating between clinically significant and insignificant prostate cancers. These studies demonstrated the clinical feasibility and relevance of time-dependent diffusion MRI. Nevertheless, none of these studies explicitly demonstrated the superiority of time-dependent diffusion MRI parameters over the conventional ADC. This is the first study to provide evidence that time-dependent diffusion MRI has additional clinical value as compared with conventional DWI.

Researchers have paid attention to the peritumoral regions in connection with the imaging differentiation of glioblastomas and brain metastases [32, 33]. Studies have investigated the use of peritumoral ADC for the differentiation of brain metastases from glioblastomas; however, its clinical value remains controversial. Lee et al. [11] reported that the minimum ADC in the peritumoral regions was useful in discriminating brain metastases from glioblastomas, whereas Tepe et al. [12] did not replicate this finding. This study revealed no significant difference in any of the peritumoral ADC indices between the two tumor types. To the best of our knowledge, time-dependent diffusion MRI has not been used to analyze peritumoral diffusion for the differentiation between brain metastases and glioblastomas. Our preliminary results suggest that ADC diffusion time dependence in the peritumoral region is not a sensitive marker for differentiating between brain metastases and glioblastomas.

Numerous studies have been published regarding MRI-based discrimination of brain metastases and glioblastomas, for which various promising structural and functional imaging parameters were reported, such as cerebral blood volume within the contrast-enhancing tumor and its surrounding areas with T2-prolongation [9] and amide proton transfer-related signal intensity in the enhancing tumor [34]. Our findings should be compared with those previous reports in future studies.

The underlying mechanism for the stronger ADC diffusion time dependence in brain metastases than in glioblastoma is unknown. In previous studies, stronger diffusion time dependence of ADC in malignant (or high-grade) tumors than in benign (or low-grade) tumors was attributed to more microstructures, which restrict water molecular motion within the range of diffusion lengths determined by the selected short and long diffusion times in the OGSE and PGSE DWI sequences, respectively [22, 23]. Although this study lacks histopathological correlation, stronger ADC diffusion time dependence in brain metastases than in glioblastomas shown in this study appears to be consistent with the hypothesized higher intracellular volume fraction in metastatic tumors than in glioblastomas. Most brain metastases originate from epithelial tumors, such as cancers of the lung, breast, and colon. Epithelial tumors are characterized by cell–cell adhesion, which, in normal tissues, determines the polarity of cells and contributes to the maintenance of tissues [35]. In contrast, glioblastomas, as nonepithelial tumors, lack cell–cell adhesion, and are characterized by microvascular proliferation and necrosis [26]. To the best of our knowledge, no data regarding the histopathological comparison of intracellular volume fraction between glioblastomas and brain metastases have been published. A recent imaging study using Vascular, Extracellular, and Restricted Diffusion for Cytometry in Tumors (VERDICT) MRI compared two metastatic brain tumors (melanomas) and five glioblastomas, and showed distinctly higher intracellular volume fraction and lower extracellular volume fraction in metastases [36]. Despite the small sample size and inclusion of only one histological type of metastasis, their results from VERDICT MRI support our speculation.

An alternative explanation for the stronger ADC diffusion time dependence in brain metastases is the difference in cell size between the two tumor types. At a given set of diffusion times (and hence diffusion length), the diffusion time dependence of ADC could vary with cell size [37]. It is possible that the cell size of brain metastases was closer to the “sweet spot” range for our diffusion time settings than that of glioblastomas. Further studies are needed to elucidate the pathological basis that accounts for our findings.

### Limitations of the study

This study has several limitations. First, the patient population was small; therefore, our suggested threshold ADC values, value of ADC difference, or relative ADC change might not be representative of those of a larger population. Second, only two diffusion times (one each for OGSE and PGSE sequences) and a fixed set of b-values (0 and 1,500 s/mm^2^) were investigated. Third, current clinical MRI systems limit OGSE to a relatively low frequency (50 Hz); therefore, the effective diffusion time was limited to 7.1 ms. Finally, we evaluated multiple types of brain metastases with a small number of each type; therefore, comparing our results using histopathology was difficult. If each tumor type is evaluated, estimating the microstructure in more detail may be possible by comparing the findings using specific histopathological features.

## Conclusions

The time-dependent diffusion MRI parameters, particularly the mean of changes in the ADC value between short and long diffusion times obtained using the OGSE and PGSE methods in the enhancing regions, may be useful in differentiating brain metastases from glioblastomas.

## Data Availability

The datasets of current study are available from the corresponding author on reasonable request.

## Abbreviations

MRI: Magnetic resonance imaging
DWI: Diffusion-weighted imaging
ADC: Apparent diffusion coefficient
PGSE: Pulsed gradient spin-echo
OGSE: Oscillating gradient spin-echo
Δ_eff_: Effective diffusion time
δ: Diffusion gradient pulse duration
Δ: Diffusion gradient separation
TR: Repetition time
TE: Echo time
FOV: Field of view
FLAIR: Fluid-attenuated inversion recovery
ROI: Region of interest
cADC: Apparent diffusion coefficient change
rcADC: Relative apparent diffusion coefficient change
ICC: Intraclass correlation coefficient
AUC: Area under the receiver operating characteristic curve
ROC: Receiver operating characteristic
